# Tailoring the Composition of HA/PEG Mixed Nano-Assemblies for Anticancer Drug Delivery

**DOI:** 10.3390/molecules30061349

**Published:** 2025-03-17

**Authors:** Beatrice Zurletti, Ilaria Andreana, Iris Chiara Salaroglio, Valeria Bincoletto, Maela Manzoli, Barbara Rolando, Paola Milla, Chiara Riganti, Barbara Stella, Silvia Arpicco

**Affiliations:** 1 Department of Drug Science and Technology, University of Turin, Via Pietro Giuria 9, 10125 Turin, Italy; beatrice.zurletti@unito.it (B.Z.); ilaria.andreana@unito.it (I.A.); valeria.bincoletto@unito.it (V.B.); maela.manzoli@unito.it (M.M.); barbara.rolando@unito.it (B.R.); paola.milla@unito.it (P.M.); barbara.stella@unito.it (B.S.); 2 Department of Oncology, Interdepartmental Center of Molecular Biotechnology “Guido Tarone”, University of Turin, Via Nizza 44, 10126 Turin, Italy; irischiara.salaroglio@unito.it (I.C.S.); chiara.riganti@unito.it (C.R.)

**Keywords:** hyaluronic acid conjugate, nano-assemblies, CD44 receptor, targeted drug delivery

## Abstract

Self-assembling amphiphilic polymers represent highly promising materials with emerging applications across various fields. In these polymers, the presence of hydrophilic and hydrophobic segments within their structure drives the self-assembly process in aqueous environments, leading to organized structures capable of incorporating lipophilic drugs. Their high chemical versatility enables the design of tailored structures to meet specific requirements, such as the active targeting ability, thereby broadening their potential applications. In this work, a polyethylene glycol-phospholipid conjugate was employed to form nanocarriers loaded with a lipophilic derivative of gemcitabine. To achieve nano-assemblies actively targeted towards cancer cells overexpressing the hyaluronic acid (HA) receptor CD44, a HA-phospholipid conjugate was co-formulated in various molar ratios (1%, 10%, and 20%). All formulations exhibited a mean diameter below 130 nm, a negative zeta potential (approximately −30 mV), and a high encapsulation efficiency (above 90%). These nano-assemblies demonstrated stability during storage and effectively released the encapsulated drug in a cell culture medium. Upon incubation with cancer cells, the nano-assemblies were internalized via a CD44 endocytosis-mediated mechanism, with the extent of internalization depending on the HA conjugate content. Consistently, cell viability studies revealed that the nanocarriers decorated with higher amounts of HA exerted a higher cytotoxicity, enabling a fine tuning of the nano-assembly properties.

## 1. Introduction

In recent decades, biocompatible amphiphilic polymers have attracted considerable attention for their ability to self-assemble in aqueous solutions into colloidal aggregates that are highly effective in drug delivery. These nanocarriers represent a promising strategy for overcoming several critical challenges in this domain: specifically, they improve the solubility of poorly water-soluble drugs and enhance their permeability across biological barriers, thereby overcoming two of the most common obstacles faced by traditional drug delivery systems [1,2]. Moreover, these polymeric nano-assemblies are particularly appealing given the ease of the preparation process, low cost, and highly adaptable nature [3].

Among the available biocompatible amphiphilic polymers, PEG-DSPE (1,2-distearoyl-*sn*-glycero-3-phosphoethanolamine-*N*-[methoxy(polyethylene glycol)]) is widely used in drug delivery applications due to its unique physicochemical properties: this conjugate combines a hydrophobic tail (the phospholipid DSPE) with a hydrophilic PEG chain, resulting in excellent self-assembly capabilities in aqueous environments to form stable nanoparticles, micelles, or liposomes [4,5]. In addition, PEG-DSPE is particularly valued for its ability to confer steric stabilization to nanocarriers, reducing opsonization and extending the circulation time in vivo [6]. However, it has been observed that the administration of PEGylated nanosystems may induce the formation of anti-PEG antibodies [7]. PEG-DSPE nano-assemblies can also be functionalized with ligands to enable active targeting to specific tissues or cells, including tumors. The targeting agent can be linked to the distal part of the PEG chain, or it can be added as a co-formulant during the nanocarrier preparation, thus avoiding further chemical modifications [4,5].

On these bases, in our previous work, we demonstrated the ability of PEG-DSPE to self-assemble in combination with hyaluronic acid (HA)-phospholipid conjugates (HA-DPPE) to form nano-assemblies able to effectively load a hydrophobic drug [8]. HA is a linear glycosaminoglycan composed of repeating units of D-glucuronic acid and N-acetyl-D-glucosamine, linked by alternating β-1,3 and β-1,4 glycosidic bonds [9]. Under physiological conditions, HA primarily exists as a sodium salt which is negatively charged and highly hydrophilic, making it widely distributed throughout the extracellular matrix [10]. Furthermore, HA exhibits excellent physicochemical properties, such as biocompatibility, biodegradability, and a lack of toxicity, immunogenicity, and inflammation [11]. Its diverse chemical groups facilitate the conjugation of various components, making HA versatile for use in nanosystems [12]. Moreover, in cancer therapy, HA is commonly employed as a targeting ligand for CD44 receptors, which are present on most tumor cells [13].

In our previous work, two HA-DPPE conjugates with different HA molecular weights (4800 and 14,800 Da) were combined in a fixed molar ratio (1%) with PEG-DSPE to form mixed nano-assemblies encapsulating a lipophilic derivative of the anticancer drug gemcitabine, 4-(*N*)-stearoylgemcitabine (GemC18), which confirmed a better antitumor efficacy than the parent drug, as already observed for other nanocarriers [8,14,15]. We also observed that the presence of 1% HA-DPPE allowed the nanocarriers to be actively targeted towards CD44-overexpressing cancer cells [8]. In particular, the formulation containing HA with the higher molecular weight (PEG-DSPE/HA_14800_-DPPE) showed to be more active against pancreatic cells [8]. Similarly, other studies showed that the HA molecular weight plays a crucial role in internalization and the simultaneous activation of different endocytic pathways of HA-coated nanoparticles by cells [16].

The aim of the present study was to vary the amount of HA_14800_-DPPE co-formulated with PEG-DSPE (increasing the molar ratio from 1% to 20%) and to evaluate the influence of this modification on the physicochemical and biological characteristics of the GemC18-loaded mixed nanocarriers. Thus, the obtained decorated nanocarriers were analyzed concerning mean size, zeta potential, drug content, drug release, X-ray powder diffraction, cell uptake, and cytotoxicity.

## 2. Results and Discussion

This study follows our previous work [8], in which PEG-DSPE nano-assemblies (NA) were prepared to improve the anticancer activity of a lipophilic gemcitabine prodrug, GemC18. Furthermore, an active targeting strategy was achieved by preparing GemC18-loaded NA in the presence of a 1% HA-DPPE conjugate with two different molecular weights (4800 and 14,800 Da). Both GemC18 and HA conjugates were synthesized in our laboratory [14,17]. PEG/HA_1%-14800_-NA were found to be more cytotoxic than PEG/HA_1%-4800_-NA on high-CD44 expressing PANC-1 cells [8]. Thus, we selected the more promising HA_14800_ conjugate for further studies; in particular, the aim of the present work was to evaluate the influence of a higher percentage (10 and 20%) of the HA_14800_-DPPE conjugate (to obtain PEG/HA_10%_-NA and PEG/HA_20%_-NA) on the physicochemical characteristics and in vitro behavior of mixed NA, which are characterized by a consequent lower amount of PEG-DSPE to maintain a fixed total molar concentration of the components. The increase of the HA content may allow for a more efficient active targeting towards cancer cells overexpressing the CD44 receptor, while a reduced PEG percentage could be preferable on the basis that the large use of PEG in various formulations, including cosmetic and food products, can cause the development of anti-PEG antibodies [7]. This phenomenon has been demonstrated to result in an accelerated blood clearance which in turn leads to a reduction in the half-life and therapeutic efficacy of PEGylated drug delivery systems [18].

### 2.1. Critical Micellar Concentration (CMC)

The ability of PEG-DSPE to self-assemble alone or in the presence of different percentages of HA_14800_-DPPE was evaluated. In particular, the exploited method was based on pyrene fluorescence, which changes its spectrum when it is organized in a hydrophobic environment. CMC was found to be about 1 × 10^−5^ M not only for PEG-NA and PEG/HA_1%_-NA, thus confirming the results previously reported [8], but also for PEG/HA_10%_-NA and PEG/HA_20%_-NA (Table S1). These findings suggest that an increase in the percentage of HA_14800_-DPPE in NA does not affect either the capacity of self-assembling or the concentration at which this phenomenon takes place.

### 2.2. Nano-Assembly Preparation and Characterization

All NA (PEG/HA_1%_-NA, PEG/HA_10%_-NA, and PEG/HA_20%_-NA) were prepared by film hydration followed by sonication [8]. In particular, an aqueous solution of HA_14800_-DPPE was used to hydrate a PEG-DSPE thin film. The suspension was then briefly sonicated. The different percentages of HA_14800_-DPPE and PEG-DSPE were achieved by tuning the amount and concentration of the component stock solutions. This is a straightforward and versatile method for the self-assembly of nanocarriers, in contrast to other techniques that involve multiple procedural steps [19].

GemC18-loaded NA (GemC18-PEG/HA_1%_-NA, GemC18-PEG/HA_10%_-NA, and GemC18-PEG/HA_20%_-NA) were obtained by adding the prodrug during the thin film formation; then, the hydration with the HA_14800_-DPPE conjugate solution allowed the prodrug to be incorporated in the inner lipophilic compartment of the NA. As GemC18 is insoluble in water, the non-associated prodrug was subsequently eliminated by filtration. Fluorescently labelled NA were prepared as well, using Nile Red as a lipophilic dye.

All NA were characterized concerning the mean hydrodynamic diameter, polydispersity index (PDI) and zeta potential. GemC18-loaded PEG/HA-NA displayed a lower mean diameter (above 50%) compared to the unloaded counterparts: this suggests that GemC18 can probably act as a surfactant during the formation of the NA thanks to its amphiphilic structure. Concerning the amount of HA_14800_-DPPE, for drug-loaded NA there is a tendency to an increase of the mean diameter with the increase of the HA_14800_-DPPE percentage; however, the mean size remains lower than 130 nm for all the formulations; thus, it is suitable for an intravenous administration. The PDI values are comprised in the 0.2–0.3 range. The zeta potential values were negative (around −30 mV) for all the formulations due to the presence of the negatively charged HA on the outer part of the NA, thus suggesting colloidal stability no matter the percentage of NA components or the presence of the prodrug (Table 1).

The amounts of phospholipids and HA in NA were measured before and after purification using the Rouser colorimetric test and the carbazole assay, respectively, to evaluate a possible loss of NA components during the purification [20,21]. The results showed a loss of phospholipids and HA of 5% for all the formulations, indicating that the purification via syringe filtration did not influence the NA component concentration.

As shown in Table 1, the EE is higher than 90% for all the GemC18-PEG/HA-NA, irrespective of the percentage of HA_14800_-DPPE, indicating that an increase in the HA_14800_-DPPE conjugate did not affect the incorporation of GemC18. On the contrary, the DL values diminished by increasing the percentage of HA_14800_-DPPE from 1% to 20%. This change in the formulation led to an important increase in the total weight of the NA; consequently, while the EE values were similar, the DL changed more noticeably due to the higher denominator value in the DL formula.

The GemC18-loaded NA stored at 4 °C did not show changes in either mean diameter and zeta potential for 21 days (an increase of less than 20% for all the formulations was observed). On the contrary, the stability studies on unloaded NA showed a significant change in mean diameter after 21 days (an increase of about 70% was observed for all the formulations), thus supporting the hypothesis of the stabilizing role of the Gem prodrug in the NA. GemC18-loaded NA stability was also tested by incubating them in Dulbecco’s Modified Eagle Medium (DMEM) with 10% fetal bovine serum (FBS) at 37 °C: no appreciable changes in mean diameter and PDI were observed for 48 h for all the loaded formulations. This result is highly significant, because, when nanocarriers are tested with cells using in vitro systems, they first interact with the components of the cell culture medium before coming into contact with the cells. The culture medium is a buffered solution mainly composed of proteins along with various molecules, including amino acids and salts. These components may affect the hydrodynamic properties of nanocarriers that may become unstable due to protein or molecule adsorption, leading to the formation of aggregates. These phenomena can further impact in vitro behavior by influencing the cellular interaction, uptake, and cytotoxicity [22,23,24]. In this work, a 48-h time interval was chosen to evaluate the change in mean size and PDI during the first phases of the interaction of the nano-assemblies with the medium before a complete drug release.

**Figure 1:**
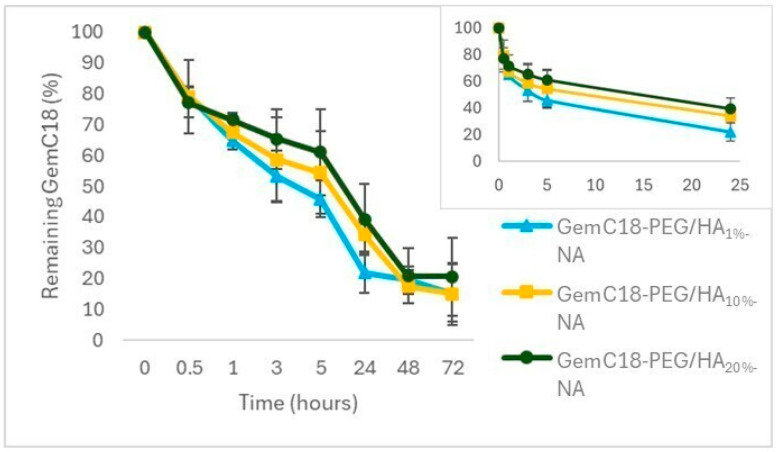
GemC18 released from GemC18-PEG/HA_1%_-NA, GemC18-PEG/HA_10%_-NA, and GemC18-PEG/HA_20%_-NA diluted 1:5 (*v*/*v*) in DMEM containing 10% of FBS as a function of time at 37 °C.

The drug release was first evaluated in phosphate saline buffer (PBS) at 37 °C. In these conditions, only GemC18-PEG/HA_1%_-NA gradually released the drug content, while for GemC18-PEG/HA_10%_-NA and GemC18-PEG/HA_20%_-NA, no free drug was detected even after 72 h (Figure S1). Thus, to verify the ability of NA with a higher HA_14800_-DPPE content to release the drug, the NA were incubated in a cell culture medium at the same temperature: in these conditions, all the formulations released the drug, with a slightly slower profile for GemC18-PEG/HA_20%_-NA compared to GemC18-PEG/HA_10%_-NA and GemC18-PEG/HA_1%_-NA (Figure 1). The presence of a higher amount of HA_14800_-DPPE in the NA structure (and, notably, of HA on the NA surface) probably hampers the release of the incorporated lipophilic drug in the buffer; however, once incubated with cells in the culture medium, the drug can be released with a similar profile for all the formulations.

**Figure 2:**
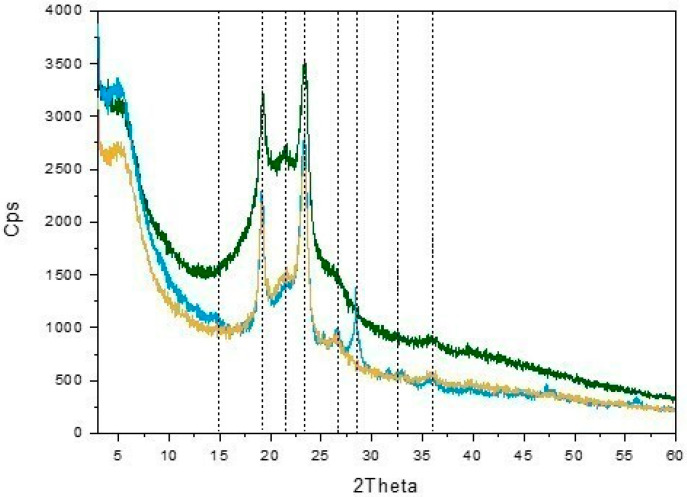
A comparison among the XRPD patterns of GemC18-PEG/HA_1%_-NA (blue line), GemC18-PEG/HA_10%_-NA (yellow line), and GemC18-PEG/HA_20%_-NA (green line). The main peaks are signaled by black dashes lines.

The structure of PEGylated NA plays a crucial role in drug delivery systems; therefore, investigating crystallinity is essential for optimizing drug solubility, release rates, stability, and bioavailability [25]. On one hand, the crystallinity of a polymeric carrier can reduce the ability to incorporate hydrophobic drugs, leading to a lower drug-loading capacity [26]. On the other hand, an increase of the amount of drug molecules inside the NA can alter the crystallinity of the carrier [27,28]. A highly crystalline carrier matrix can be beneficial for controlled drug delivery, because drug diffusion out of the system may be hindered, leading to a slow and sustained release profile [5]. As a matter of fact, crystallinity enhances structural integrity resulting in improved stability and enhanced encapsulation efficiency. Thus, the effect of the amount of the HA_14800_-DPPE conjugate on the structure of GemC18-PEG/HA_1%_-NA, GemC18-PEG/HA_10%_-NA, and GemC18-PEG/HA_20%_-NA was investigated by XRPD (Figure 2). These samples are designed with a decreasing PEG content. In addition to the signal at 2Theta = 4.9° previously ascribed to the presence of NA, the peaks due to the presence of crystalline PEG (JCPDS file number 00-049-2095) were observed in all the patterns at 2Theta = 15.0°, 19.2°, 21.9°, 23.2°, and 26.8°. More in detail, the peaks at 2Theta = 19.2° and 23.2° have been ascribed either to the (115) and (016) [29] or (120) and (032) lattice planes [30]. Interestingly, the intensity (I) ratio between these two peaks (I23.2°/I19.2°) decreased from 1.41 for GemC18-PEG/HA_10%_-NA to 1.25 for GemC18-PEG/HA_20%_-NA, likely indicating that when increasing the HA content, the HA_14800_-DPPE conjugate interacts with PEG-DSPE preferentially along the direction defined by the 23.3° peak.

**Figure 3:**
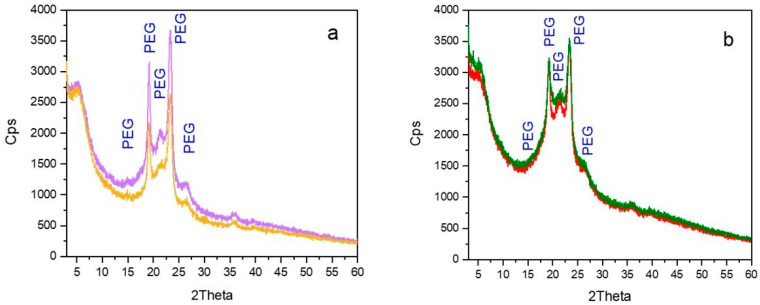
A comparison between the PXRD patterns of (**a**) PEG/HA_1%_-NA (violet line) and GemC18-PEG/HA_10%_-NA (yellow line) and (**b**) PEG/HA_20%_-NA (red line) and GemC18-PEG/HA_20%_-NA (green line). The main peaks related to PEG are signaled.

Moreover, the peak at 2Theta 28.4°, previously assigned to PEG modified by the conjugation with DSPE [8], was detected in the case of GemC18-PEG/HA_1%_-NA (blue line), whereas it drastically decreased in intensity and disappeared (yellow line and green line, respectively) as a consequence of the HA_14800_-DPPE percentage increase and the corresponding decrease of the amount of PEG-DSPE in the NA. Overall, the XRPD experiments clearly demonstrated that increasing the amount of HA_14800_-DPPE conjugate did not affect the overall crystallinity of the NA. This agrees with the results shown in Figure 1 which indicated a higher GemC18 retention by increasing the HA percentage and decreasing the PEG-DSPE content. The same trend was also observed in the absence of GemC18 (Figure 3), also indicating that enhancing the HA_14800_-DPPE content did not compromise the insertion of GemC18 in the NA.

### 2.3. Hemolysis Assay

To explore the blood compatibility of NA in view of an intravenous administration, a hemolysis test on fresh human blood was conducted with empty and GemC18-loaded PEG/HA-NA with different HA_14800_-DPPE percentages. No hemolytic activity (hemolysis < 2% for all samples) was observed at a NA concentration ranging from 0.70 μg/mL to 0.13 mg/mL for both blank and drug-loaded NA.

### 2.4. Biological Validation Assay

**Figure 4:**
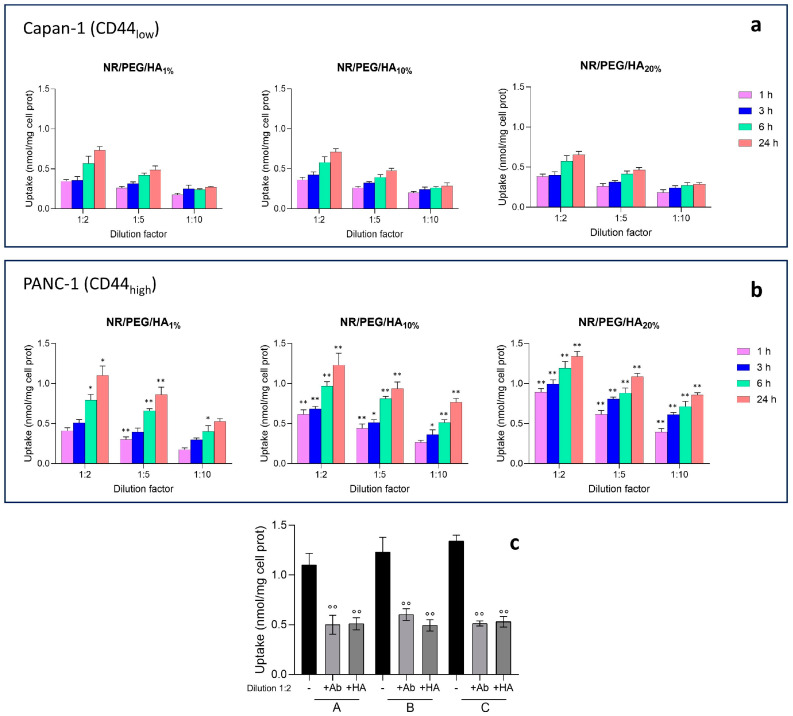
The cellular uptake by Capan-1 (**a**) and PANC-1 (**b**) cells of NA (samples diluted 1:2 (*v*/*v*), 1:5 (*v*/*v*), and 1:10 (*v*/*v*) with the culture medium), labelled with Nile Red (NR), after 1, 3, 6, or 24 h. The autofluorescence of untreated cells was subtracted from each value. Data are presented as the means ± SD (*n* = 3). * *p* < 0.1, ** *p* < 0.01: vs. untreated cells. For all the statistical comparisons see Table S2. (**c**) PANC-1 cells were treated with NA (A: NR/PEG/HA_1%_-NA, B: NR/PEG/HA_10%_-NA, and C: NR/PEG/HA_20%_-NA) diluted 1:2 for 24 h, in the absence (-) or presence of the anti-CD44 antibody diluted 1/100 (Ab) and HA (100 μM). Data are presented as the means ± SD (*n* = 3). °° *p* < 0.01: HA/Ab-treated NR/PEG/HA_1%_-NA or NR/PEG/HA_10%_-NA or NR/PEG/HA_20%_-NA vs. NR/PEG/HA_1%_-NA or NR/PEG/HA_10%_-NA or NR/PEG/HA_20%_-NA.

Nile Red fluorescently labelled PEG/HA-NA (NR/PEG/HA_1%_-NA, NR/PEG/HA_10%_-NA, and NR/PEG/HA_20%_-NA) were incubated with Capan-1 and PANC-1 cells to evaluate the NA uptake as a function of the HA_14800_-DPPE percentage. In Capan-1 cells, which express a low amount of the CD44 receptor, a time- and dose-dependent uptake of NA was observed, as indicated by the amount of Nile Red retained inside the cells (Figure 4a). A significant difference between the different formulations was not observed. As expected, the presence of the blocking anti-CD44 antibody or the excess of HA did not reduce the uptake of the NA, given the low amount of CD44. These results suggest that the uptake of the NA cargo was due to passive diffusion. Also, in the highly CD44-expressing PANC-1 cells, the uptake of NA followed a time- and dose-dependent trend, but the amount of the dye reached intracellularly was higher compared to the corresponding experimental condition of Capan-1 cells (Figure 4b). This data, together with the abrogation of the uptake in the presence of the anti-CD44 antibody or HA saturating amounts (Figure 4c), suggested that the accumulation of the NA was partly due to passive diffusion (as in the case of Capan-1 cells), partly mediated by a CD44 endocytosis-mediated mechanism. Intriguingly, the amount of Nile Red retained within PANC-1 cells was higher when the amount of HA_14800_-DPPE increased, following this rank order: NR/PEG/HA_1%_-NA < NR/PEG/HA_10%_-NA < NR/PEG/HA_20%_-NA, indicating that the higher the amount of HA that was on the NA surface, the higher the internalization was via the CD44 receptor.

**Figure 5:**
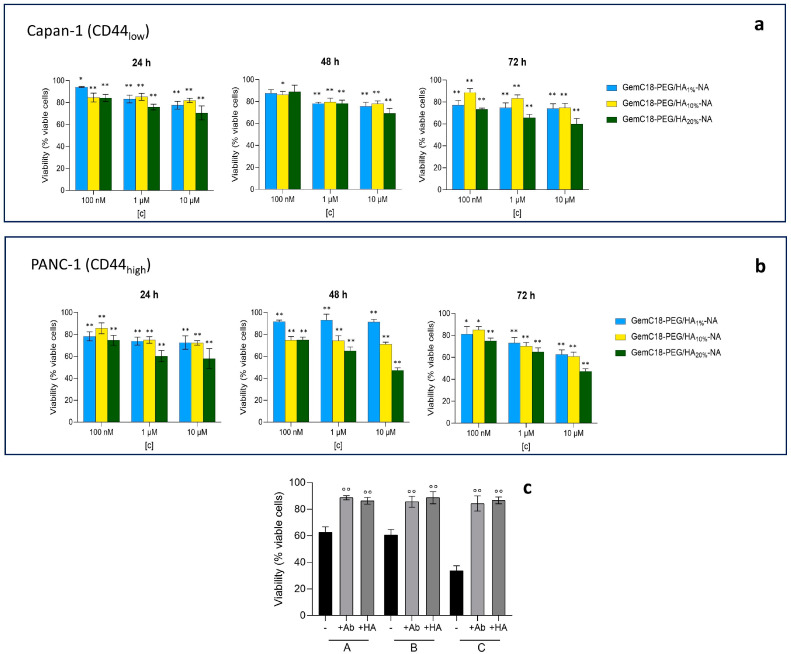
The cell viability of Capan-1 (**a**) and PANC-1 (**b**) cells, incubated with GemC18-PEG/HA_1%_-NA or GemC18-PEG/HA_10%_-NA or GemC18-PEG/HA_20%_-NA, containing different concentrations (100 nM, 1 μM, and 10 μM) of GemC18, for 24, 48, or 72 h. The cell viability of untreated cells was 100% for all experimental conditions. Data are presented as the means ± SD (*n*= 3). * *p* < 0.1, ** *p* < 0.01: vs. untreated cells; for all the statistical comparisons see Table S3. (**c**) PANC-1 cells were grown for 72 h in fresh medium (-) or in the presence of GemC18-PEG/HA_1%_-NA (A) or GemC18-PEG/HA_10%_-NA (B) or GemC18-PEG/HA_20%_-NA (C) containing 10 μM GemC18, in the absence (-) or presence of HA (100 μM) or the anti-CD44 antibody diluted 1/100 (Ab), then cell viability was measured. Data are presented as the means ± SD (*n*= 3). °° *p* < 0.01: HA/Ab-treated GemC18-PEG/HA_1%_-NA or GemC18-PEG/HA_10%_-NA or GemC18-PEG/HA_20%_-NA vs. GemC18-PEG/HA_1%_-NA or GemC18-PEG/HA_10%_-NA or GemC18-PEG/HA_20%_-NA.

When loaded with GemC18, the cell viability was time- and dose-dependent in Capan-1 (Figure 5a) as well as in PANC-1 (Figure 5b) cells. Also in this case, the NA decorated with a higher amount of HA exerted a higher cytotoxicity: the effects were detected in both cell lines, but it was more pronounced in PANC-1 cells. In these cells, the viability was mediated by the endocytosis via the CD44 receptor, since even at the highest concentration of GemC18, the cytotoxicity was abrogated by the presence of the anti-CD44 antibody or HA (Figure 5c), differently from Capan-1 cells. The differences in cell viability between Capan-1 and PANC-1, exerted by the same type of HA-decorated NA, carrying the same amount of Gem18 and incubated for the same time, may provide a measure of the role that HA decoration has in facilitating the delivery of GemC18 via CD44 instead of that by simple diffusion.

In this work, we decided to use PEG-DSPE as the main copolymer, as previously reported for other self-assembled nanosystems for GemC18 delivery [31]. Specifically, the modification of gemcitabine with an alkyl chain similar to that in DSPE promotes hydrophobic interactions and a high loading capacity. HA conjugates have been extensively explored to formulate self-assembled drug delivery systems. Specifically, HA-phospholipids derivatives, such as HA-DMPE and HA-DSPE, were employed as the primary matrix for paclitaxel delivery [32]. In our approach, we used an HA-DPPE conjugate to anchor the phospholipid to the PEG-DSPE lipid matrix during NA formation and the subsequent incorporation of GemC18, thereby establishing an outer HA shell without compromising the self-assembly process. We systematically investigated the impact of varying HA-DPPE conjugate percentages within the NA matrix, in contrast to other studies that did not optimize the percentage of the HA conjugate in the formulation [33,34]. Across formulations containing 1 to 20% HA-DPPE, no significant changes in the physicochemical properties of NA were observed. Notably, NA exhibited a high drug loading capacity, sustained drug release kinetics, and enhanced tumor targeting through an improved cellular uptake. These findings underscore the critical role of optimizing HA decoration in addressing the lack of targeting moieties in the PEG-DSPE matrix. Determining the optimal HA surface modification is essential for achieving a balance between receptor-mediated recognition, physicochemical stability, and minimizing premature drug release.

A potential intravenous administration of these nanosystems is expected to preserve drug potency while enhancing specificity toward CD44-expressing tissues. However, while CD44 expression in cancer cells facilitates the endocytosis of HA-based nanosystems, its presence in healthy tissues may lead to off-target effects. Previous studies have reported that disrupting HA-rich tumor stroma using PEGylated recombinant human hyaluronidase (PEGPH20) resulted in severe adverse effects, including musculoskeletal toxicity and thromboembolism [35]. Unlike enzymatic degradation strategies, the NA proposed in this study are designed to exploit the preferential uptake by CD44-overexpressing cells also promoting the enhanced permeation and retention effect, as previously demonstrated with other HA-based nanosystems [36,37]. Furthermore, considering the clinical advancements of HA-drug conjugates [38], optimized HA-based formulations hold promise for achieving selective tumor accumulation while minimizing systemic toxicity.

## 3. Materials and Methods

### 3.1. Materials and General Procedures

Sodium hyaluronate (HA) with a molecular weight (MW) of 14,800 Da was obtained from Lifecore Biomedical (Chaska, MN, USA). 1,2-distearoyl-*sn*-glycero-3-phosphoethanolamine-*N*-[methoxy(polyethylene glycol)-2000] (ammonium salt) (PEG-DSPE) and 1,2-dipalmitoyl-*sn*-glycero-3-phosphoethanolamine (DPPE) were purchased from Avanti Polar-Lipids, distributed by Merck Life Science (Milan, Italy). Pyrene, 0.45 and 0.22 μm nylon filters, fetal bovine serum (FBS), and solvents were obtained by Merck Life Science. DMEM and RPMI-1640 were from Invitrogen Life Technology (Milan, Italy). Solvent evaporation was performed in a rotary evaporator (Heidolph Laborota 400, Heidolph Instruments, Schwabach, Germany) equipped with a vacuum pump (Diaphragm Vacuum Pump DC-4). The HA_14800_-DPPE conjugate was synthesized following the method described in Arpicco et al. [17] and GemC18 was synthesized using the method described in Immordino et al. [14].

### 3.2. Critical Micellar Concentration

The CMC of PEG-DSPE alone or with HA_14800_-DPPE, testing different molar ratios, was measured using pyrene as a fluorescent probe by fluorescence spectroscopy [39]. Briefly, 50 µL aliquots of a 2.00 × 10^−5^ M pyrene stock solution in acetone were placed in a series of glass tubes and the solvent was evaporated under reduced pressure using rotary evaporation. A final pyrene concentration of 2.00 × 10^−6^ M was achieved by adding different aqueous solutions of PEG-DSPE alone or with HA_14800_-DPPE (1%, 10%, or 20% relative to PEG-DSPE) at concentrations ranging from 1.00 × 10^−8^ M to 1.27 × 10^−3^ M. Each sample was then incubated at 60 °C for 20 min followed by equilibration at room temperature overnight. Before the analysis, each sample was filtrated through a 0.22 μm nylon syringe filter and the fluorescence spectra of each sample were recorded using an EnSight HH3400 spectrofluorometer (PerkinElmer, Inc., Waltham, MA, USA) equipped with a Kaleido 1.2 data recorder. The excitation wavelength was 339 nm and the emission spectra were analyzed from 360 to 450 nm. The excitation slit widths were set at 373, 384, and 390 nm. The results were expressed as the correlation between log[phospholipid] and the intensity ratio of either I373/I384 or I390.

### 3.3. Preparation of the Nano-Assemblies

PEG/HA nano-assemblies (PEG/HA-NA) at different PEG/HA molar ratios (1%, 10%, and 20% of HA_14800_-DPPE compared to PEG-DSPE) were prepared using the method previously described by Andreana et al. [8], with minor modifications. Briefly, for PEG/HA_1%_-NA, 2 mg (7.13 × 10^−7^ mol) of PEG-DSPE were dissolved in 200 μL of chloroform and then evaporated under reduced pressure to obtain a thin film. Subsequently, the film was hydrated with 3 mL of an aqueous solution of HA_14800_-DPPE 0.037 mg/mL (7.13 × 10^−9^ mol). The obtained suspension was incubated at 60 °C for 10 min, then allowed to cool and sonicated in an ice bath using a VCX400 probe sonicator (Sonics & Materials Inc., Milan, Italy) for 10 min with 3 sec on/off pulses.

The same procedure was followed for PEG/HA_10%_-NA and PEG/HA_20%_-NA: practically, for PEG/HA_10%_-NA, the thin film, containing 1.8 mg (6.42 × 10^−7^ mol) of PEG-DSPE was hydrated with 3 mL of an aqueous solution of HA_14800_-DPPE 0.37 mg/mL (7.13 × 10^−8^ mol), while for PEG/HA_20%_-NA, the thin film containing 1.6 mg (5.70 × 10^−7^ mol) of PEG-DSPE was hydrated with 3 mL of an aqueous solution of HA_14800_-DPPE 0.73 mg/mL (1.43 × 10^−7^ mol).

For fluorescently labelled PEG/HA-NA (NR/PEG/HA_1%_-NA, NR/PEG/HA_10%_-NA, and NR/PEG/HA_20%_-NA), during the film preparation 40 μL of a Nile Red solution (200 μg/mL in dichloromethane) were added in each formulation and then the previously described method was followed.

For GemC18-loaded nano-assemblies (GemC18-PEG/HA_1%_-NA, GemC18-PEG/HA_10%_-NA, and GemC18-PEG/HA_20%_-NA), 500 μL of a Gem18 solution 1 mg/mL in methanol were added during the PEG-DSPE film preparation; then, nano-assemblies were obtained as described before. The drug-loaded nano-assemblies were purified from unloaded GemC18 by filtration through 0.22 μm nylon syringe filters.

### 3.4. Physicochemical Characterization of the Nano-Assemblies

The mean particle size and polydispersity index (PDI) of the different nano-assemblies samples were measured at 25 °C by quasi-elastic light scattering (QELS) using a nanosizer (Zetasizer Pro, Malvern Inst., Malvern, UK) at a detection angle of 173°. The measurement was performed on undiluted formulations and each sample was analyzed in triplicate. The surface charge of the formulations was assessed by a zeta potential measurement at 25 °C using the Smoluchowski equation and the Zetasizer Pro directly on the pure samples. Each reported value represents the average of three measurements. The concentration of the phospholipids and HA_14800_-DPPE in the nano-assemblies was evaluated before and after purification; in particular, the phospholipids were quantified by the colorimetric assay of Rouser, while HA by the carbazole assay [20,21].

The encapsulation efficiency (EE) and drug loading (DL) of GemC18-loaded nano-assemblies were determined by HPLC following the method previously described in Andreana et al. [8]. In particular, GemC18 EE was calculated following the equation:

EE%=associated drug amounttotal drug amount×100

The DL was calculated as follows:

DL%=associated drug weighttotal NA weight×100

The physical stability of the nano-assemblies in storage conditions (4 °C) was determined by evaluating the mean diameter and the zeta potential at 0, 7, 14, 21, and 28 days after preparation. The physical stability of the formulations was also evaluated in a cell culture medium by diluting the samples 1:10 (*v*/*v*) in DMEM containing 10% FBS and keeping the samples at 37 °C for 1, 24, and 48 h.

### 3.5. In Vitro Release Study

The release of GemC18 from the nano-assemblies was assessed either in buffer or in the cell culture medium following the method previously described by Andreana et al. [8]. Briefly, for buffer, 3 mL of each sample (GemC18-PEG/HA_1%_-NA, GemC18-PEG/HA_10%_-NA, and GemC18-PEG/HA_20%_-NA) were assessed in a dialysis bag immersed in 300 mL of PBS 1 mM (pH 7.4) and aliquots of 100 μL were collected at specified time intervals (0, 0.5, 1, 3, 5, 24, 48, and 72 h). For the cell culture medium, the samples (GemC18-PEG/HA_1%_-NA, GemC18-PEG/HA_10%_-NA, and GemC18-PEG/HA_20%_-NA) were diluted 1:5 (*v*/*v*) in DMEM containing 10% of FBS and incubated at 37 °C. At several time points (0, 0.5, 1, 3, 5, 24, 48, and 72 h), aliquots of the formulation were withdrawn and purified via syringe filtration through a 0.45 μm nylon filter. All the aliquots were then analyzed using the HPLC method previously described.

### 3.6. X-Ray Powder Diffraction Measurements

X-Ray Powder Diffraction (XPRD) patterns of the lyophilized nano-assemblies with different HA percentages were acquired in the 3° ≤ 2θ ≤ 60° angular range, with 0.02° 2θ steps by a PW3050/60 X’Pert PRO MPD diffractometer from PANalytical (Almelo, Netherlands) working in Bragg–Brentano geometry, employing a high-powered ceramic tube PW3373/10 LFF as the source with a Cu anode (Cu K_α1_ radiation λ = 1.5406 Å) equipped with a Ni filter to attenuate K_β_. Scattered photons were collected by a real-time multiple strip (RTMS) X’celerator detector. The samples in the form of powders were examined in their as-prepared form using a spinning sample holder to minimize the preferred orientations. Nano-assemblies without GemC18, i.e., PEG/HA_1%_-NA, PEG/HA_10%_-NA, and PEG/HA_20%_-NA, were also measured as reference samples.

### 3.7. Hemolysis Assay

A hemolysis test was conducted on all PEG/HA-NA and GemC18-loaded PEG/HA-NA. Practically, an erythrocytes suspension 3% (*w*/*w*) in saline was prepared using fresh human blood. The blood used for the hemolysis experiments was obtained from a healthy voluntary donor under an agreement between the Department of Oncology of the University of Turin and the Blood Bank of the AOU Città della Salute di Torino (protocol no. 993/2023). The donor provided informed consent in compliance with current regulations (Declaration of Helsinki, GDPR). Nano-assemblies at different phospholipid concentrations ranging from 0.70 μg/mL to 0.13 mg/mL were mixed with a 150 μL aliquot of the erythrocyte suspension for a total volume of 300 μL. The negative and positive control were a saline solution and deionized water, respectively. All the samples were incubated at 37 °C for 1 h under constant magnetic stirring. The suspension was then centrifuged at 2500× *g* for 5 min to remove intact erythrocytes. The supernatant was collected and analyzed for released hemoglobin using an EnSight (Lubbock, TX, USA) HH3400 spectrofluorometer at 540 nm. The degree of hemolysis was calculated as follows:

$$Hemolysis\left(\%\right)=\frac{Am-Aneg}{Apos-Aneg}\times 100$$

where Am is the absorbance of nano-assemblies, and Apos and Aneg are the absorbance of a 100% hemolyzed solution and of a 0% hemolyzed solution [40].

### 3.8. Cell Lines

Human pancreatic adenocarcinoma cells, Capan-1 and PANC-1, purchased from ATCC (Manassas, VA, USA), were cultured in DMEM (Capan-1) and RPMI-1640 (PANC-1) media, with 1% *v*/*v* penicillin–streptomycin and 10% FBS (Merck Life Science). According to the surface amount of the HA receptor CD44, evaluated by flow cytometry as in [41], Capan-1 and PANC-1 cells were classified as CD44-low and -high expressing cells, respectively [8].

### 3.9. Nano-Assemblies Uptake

A total of 1 × 10^5^ cells, seeded into a 96-well plate in a 200 µL medium, were incubated for 1, 3, 6, and 24 h with free Nile Red or Nile Red-loaded nano-assemblies (NR/PEG/HA_1%_-NA, NR/PEG/HA_10%_-NA, and NR/PEG/HA_20%_-NA) after diluting the samples 1:2 (*v*/*v*), 1:5 (*v*/*v*), and 1:10 (*v*/*v*) with the culture medium. In competition assays, a saturating amount of the blocking anti-CD44 antibody (#ab157107; Abcam, Cambridge, UK; diluted 1/100) or HA (100 μM) was added to the cells incubated with nano-assemblies diluted 1:2 (*v*/*v*) for 24 h. The nano-assemblies uptake was quantified fluorimetrically as reported in Andreana et al. [8]. The fluorescence of untreaded cells was measured and subtracted as blank in each experimental set.

### 3.10. Cell Viability

A total of 1 × 10^4^ cells were seeded into a 96-well white plate in 200 µL medium and incubated for 24, 48, and 72 h with either the fresh medium, or medium containing GemC18-PEG/HA_1%_-NA, GemC18-PEG/HA_10%_-NA, or GemC18-PEG/HA_20%_-NA in the range of the concentration of GemC18 from 0.1 nM to 10 µM. In the competition assays, cells exposed to the highest NA concentration (corresponding to 10 µM GemC18) for 72 h were co-incubated with the blocking anti-CD44 antibody diluted 1/100 or HA (100 μM). Cell viability was measured by a chemiluminescence-based assay as reported in Andreana et al. [8].

### 3.11. Statistical Analysis

Data in the text and figures are provided as the means ± SD. The results were analyzed by a one-way analysis of variance (ANOVA) and Tukey’s test. *p* < 0.05 was considered significant.

## 4. Conclusions

This work aimed to evaluate the influence of different amounts of the amphiphilic conjugate HA_14800_-DPPE on the characteristics of drug-loaded PEG-DSPE NA. More in detail, 1%, 10%, and 20% HA_14800_-DPPE were added to PEG-DSPE during NA preparation and a lipophilic prodrug of gemcitabine, GemC18, was entrapped into the inner NA core, resulting in a decorated nanocarrier obtained through a straightforward and versatile procedure. Despite a slight increase in the mean diameter as a function of the HA_14800_-DPPE amount, all NA displayed a size below 130 nm, a negative zeta potential, and a high EE. GemC18 showed a stabilization of the formulations, irrespective of the amount of HA_14800_-DPPE; furthermore, the drug was stably associated with the NA, which released it only in the cell culture medium. In vitro experiments demonstrated that HA-coated NA exhibited an improved cellular uptake and increased toxicity on cancer cells with high levels of the CD44 receptor in comparison to their uncoated counterparts. This result was even more evident for NA with higher HA_14800_-DPPE content.

The next steps will concern the analysis of the NA shape for the different HA_14800_-DPPE percentages. Furthermore, the ability of the NA decorated with high amounts of HA to actively target cancer cells in vivo will be evaluated.

## Figures and Tables

**Table 1:** The mean diameter, polydispersity index (PDI), zeta potential, encapsulation efficiency (EE), and drug loading (DL) of GemC18-loaded PEG/HA-NA.

Sample	Mean Diameter (nm ± S.D.)	PDI	Zeta Potential (mV ± S.D.)	EE (% ± S.D.)	DL (% ± S.D.)
GemC18-PEG/HA_1%_-NA	68 ± 10	0.272	−27 ± 8	96 ± 3	18 ± 2
GemC18-PEG/HA_10%_-NA	97 ± 10	0.290	−30 ± 5	91 ± 9	13 ± 2
GemC18-PEG/HA_20%_-NA	122 ± 24	0.304	−27 ± 6	92 ± 8	8 ± 2

## Data Availability

The raw data supporting the conclusions of this article will be made available by the authors on request.

## Supplementary Materials

The following supporting information can be downloaded at https://www.mdpi.com/article/10.3390/molecules30061349/s1; Table S1. Critical micellar concentration (CMC) for the PEG/HA mixed nanoassemblies. Table S2. Results of the statistical analysis. Table S3. Results of the statistical analysis. Figure S1. GemC18 release from GemC18-PEG/HA_1%_-NA, GemC18-PEG/HA_10%_-NA and GemC18-PEG/HA_20%_-NA as a function of time in PBS pH 7.4 at 37 °C.
