# Supplementary material for: Tailoring the Composition of HA/PEG Mixed Nano-Assemblies for Anticancer Drug Delivery
Source: Molecules. 2025 Mar 17;30(6):1349. doi: 10.3390/molecules30061349 (PMC11945053; doi:10.3390/molecules30061349)
Supplement: Supplementary file 1 [file molecules-30-01349-s001.zip › molecules-3486414-supplementary.pdf]

Supplementary Materials

Tailoring the Composition of HA/PEG Mixed Nano- Assemblies for Anticancer Drug Delivery

Table S1. Critical micellar concentration (CMC) for the PEG/HA mixed nanoassemblies.

Sample	CMC (M)
PEG/$HA_{1\%}$-NA	1.0×10^{-5}
PEG/$HA_{10\%}$-NA	0.9×10^{-5}
PEG/$HA_{20\%}$-NA	0.8×10^{-5}

Table S2. Results of the statistical analysis. *p<0.1, **p<0.01, ***p<0.001, ****p<0.00001.

Uptake Panc-1 NR/PEG/$HA_{1\%}$	Sample	Significance
**1:2**	1 h *vs* 6 h	****
	1 h *vs* 24 h	****
	3 h *vs* 6 h	****
	3 h *vs* 24 h	****
	6 h *vs* 24 h	****
	3 h *vs* 24 h	****
	6 h *vs* 24 h	**
**1:5**	1 h *vs* 6 h	****
	1 h *vs* 24 h	****

	3 h *vs* 6 h	****
	3 h *vs* 24 h	****
	6 h *vs* 24 h	**
**1:10**	1 h *vs* 6 h	***
	1 h *vs* 24 h	****
	3 h *vs* 24 h	***
**Uptake Panc-1 NR/PEG/$HA_{10\%}$**		
**1:2**	1 h *vs* 6 h	****
	1 h *vs* 24 h	****
	3 h *vs* 6 h	****
	3 h *vs* 24 h	****
	6 h *vs* 24 h	***
**1:5**	1 h *vs* 6 h	****
	1 h *vs* 24 h	****
	3 h *vs* 6 h	****
	3 h *vs* 24 h	****
**1:10**	1 h *vs* 6 h	***
	1 h *vs* 24 h	****
	3 h *vs* 6 h	*
	3 h *vs* 24 h	****
	6 h *vs* 24 h	***
**Uptake Panc-1 NR/PEG/$HA_{20\%}$**		
**1:2**	1 h *vs* 6 h	****
	1 h *vs* 24 h	****
	3 h *vs* 6 h	***
	3 h *vs* 24 h	****
	6 h *vs* 24 h	**
**1:5**	1 h *vs* 3 h	***
	1 h *vs* 6 h	****
	1 h *vs* 24 h	****

	3 h *vs* 24 h	****
	6 h *vs* 24 h	***
**1:10**	1 h *vs* 3 h	***
	1 h *vs* 6 h	****
	1 h *vs* 24 h	****
	3 h *vs* 24 h	****
	6 h *vs* 24 h	*
**Uptake Capan-1 NR/PEG/HA$_{1\%}$**		
**1:2**	1 h *vs* 6 h	****
	1 h *vs* 24 h	****
	3 h *vs* 6 h	****
	3 h *vs* 24 h	****
	6 h *vs* 24 h	***
**1:5**	1 h *vs* 6 h	***
	1 h *vs* 24 h	****
	3 h *vs* 6 h	*
	3 h *vs* 24 h	***
**1:10**	1 h *vs* 24 h	*
**Uptake Capan-1 NR/PEG/HA$_{10\%}$**		
**1:2**	1 h *vs* 6 h	****
	1 h *vs* 24 h	****
	3 h *vs* 6 h	****
	3 h *vs* 24 h	****
	6 h *vs* 24 h	***
**1:5**	1 h *vs* 6 h	***
	1 h *vs* 24 h	****
	3 h *vs* 24 h	***
	6 h *vs* 24 h	*
**1:10**	1 h *vs* 24 h	*

**Uptake Capan-1 NR/PEG/HA$_{20\%}$**		
**1:2**	1 h *vs* 6 h	****
	1 h *vs* 24 h	****
	3 h *vs* 6 h	****
	3 h *vs* 24 h	****
**1:5**	1 h *vs* 6 h	***
	1 h *vs* 24 h	****
	3 h *vs* 6 h	*
	3 h *vs* 24 h	***
**1:10**	1 h *vs* 6 h	*
	1 h *vs* 24 h	*

Table S3. Results of the statistical analysis. * $p < 0.1$, ** $p < 0.01$, *** $p < 0.001$, **** $p < 0.00001$.

**Viability Panc-1 24 h**	**Sample**	**Significance**
**100 nM**	GemC18-PEG/HA$_{10\%}$-NA *vs* GemC18-PEG/HA$_{20\%}$-NA	*
**1 µM**	GemC18-PEG/HA$_{1\%}$-NA *vs* GemC18-PEG/HA$_{20\%}$-NA	*
	GemC18-PEG/HA$_{10\%}$-NA *vs* GemC18-PEG/HA$_{20\%}$-NA	**
**10 µM**	GemC18-PEG/HA$_{1\%}$-NA *vs* GemC18-PEG/HA$_{20\%}$-NA	**
	GemC18-PEG/HA$_{10\%}$-NA *vs* GemC18-PEG/HA$_{20\%}$-NA	**
**Viability Panc-1 48 h**		
**100 nM**	GemC18-PEG/HA$_{1\%}$-NA *vs* GemC18-PEG/HA$_{10\%}$-NA	****
	GemC18-PEG/HA$_{1\%}$-NA *vs* GemC18-PEG/HA$_{20\%}$-NA	****
**1 µM**	GemC18-PEG/HA$_{1\%}$-NA *vs* GemC18-PEG/HA$_{10\%}$-NA	****
	GemC18-PEG/HA$_{1\%}$-NA *vs* GemC18-PEG/HA$_{20\%}$-NA	**
	GemC18-PEG/HA$_{10\%}$-NA *vs* GemC18-PEG/HA$_{20\%}$-NA	**
**10 µM**	GemC18-PEG/HA$_{1\%}$-NA *vs* GemC18-PEG/HA$_{10\%}$-NA	****
	GemC18-PEG/HA$_{1\%}$-NA *vs* GemC18-PEG/HA$_{20\%}$-NA	****
	GemC18-PEG/HA$_{10\%}$-NA *vs* GemC18-PEG/HA$_{20\%}$-NA	****

**Viability Panc-1 72 h**		
**100 nM**	GemC18-PEG/HA$_{10\%}$-NA *vs* GemC18-PEG/HA$_{20\%}$-NA	*
**10 µM**	GemC18-PEG/HA$_{1\%}$-NA *vs* GemC18-PEG/HA$_{20\%}$-NA	***
	GemC18-PEG/HA$_{10\%}$-NA *vs* GemC18-PEG/HA$_{20\%}$-NA	**

**Viability Capan-1 24 h**		
**100 nM**	GemC18-PEG/HA$_{1\%}$-NA *vs* GemC18-PEG/HA$_{10\%}$-NA	**
	GemC18-PEG/HA$_{1\%}$-NA *vs* GemC18-PEG/HA$_{20\%}$-NA	**
**1 µM**	GemC18-PEG/HA$_{10\%}$-NA *vs* GemC18-PEG/HA$_{20\%}$-NA	*
**10 µM**	GemC18-PEG/HA$_{10\%}$-NA *vs* GemC18-PEG/HA$_{20\%}$-NA	**
**Viability Capan-1 48 h**		
**10 µM**	GemC18-PEG/HA$_{10\%}$-NA *vs* GemC18-PEG/HA$_{20\%}$-NA	*
**Viability Capan-1 72 h**		
**100 nM**	GemC18-PEG/HA$_{1\%}$-NA *vs* GemC18-PEG/HA$_{10\%}$-NA	**
	GemC18-PEG/HA$_{10\%}$-NA *vs* GemC18-PEG/HA$_{20\%}$-NA	***
**1 µM**	GemC18-PEG/HA$_{1\%}$-NA *vs* GemC18-PEG/HA$_{10\%}$-NA	*
	GemC18-PEG/HA$_{1\%}$-NA *vs* GemC18-PEG/HA$_{20\%}$-NA	*
	GemC18-PEG/HA$_{10\%}$-NA *vs* GemC18-PEG/HA$_{20\%}$-NA	****
**10 µM**	GemC18-PEG/HA$_{1\%}$-NA *vs* GemC18-PEG/HA$_{20\%}$-NA	***
	GemC18-PEG/HA$_{10\%}$-NA *vs* GemC18-PEG/HA$_{20\%}$-NA	***

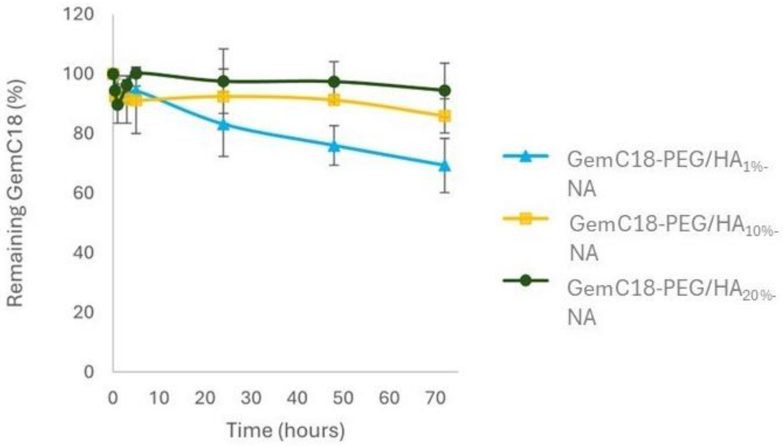

Figure S1. GemC18 release from GemC18-PEG/HA$_{1\%}$-NA, GemC18-PEG/HA$_{10\%}$-NA and GemC18-PEG/HA$_{20\%}$-NA as a function of time in PBS pH 7.4 at 37 °C.
